# Risk factor of postoperative adverse events among children with duplex kidney undergoing upper pole heminephrectomy: a single-center experience

**DOI:** 10.3389/fped.2024.1305456

**Published:** 2024-04-29

**Authors:** Denghui Wang, Mengjie Cui, Xiangyang Chu, Xiaojiang Han, Pengpeng Liu, Xiang Zhao, Yingzhong Fan

**Affiliations:** Department of Pediatric Urology, The First Affiliated Hospital of Zhengzhou University, Zhengzhou, China

**Keywords:** children, duplex kidney, heminephrectomy, risk factors, analysis

## Abstract

**Objective:**

The aim of this study was to identify the risk factors for postoperative adverse events in children with duplex kidney undergoing upper pole heminephrectomy.

**Methods:**

We collected clinical data from pediatric patients with duplex kidney who underwent upper pole heminephrectomy. Based on the presence or absence of postoperative adverse events, the patients were divided into two groups: an adverse events group (*n* = 16) and a non- adverse events group (*n* = 37), using multivariate logistic regression analysis to screen for independent risk factors for postoperative adverse events.

**Results:**

Through univariate and multivariate analysis, we found that the presence of upper renal ureterocele (*P* = 0.042, OR = 7.116, 95% CI 1.073–47.172), as well as the presence of accessory renal artery type (*P* = 0.016, OR = 10.639, 95% CI 1.551–72.978) and other types (*P* = 0.039, OR = 3.644, 95% CI 0.351–37.836) as the upper kidney's blood supply artery increase the risk of postoperative adverse events, with these differences being statistically significant.

**Conclusions:**

In pediatric patients with duplex kidney undergoing upper pole heminephrectomy, the presence of upper renal ureterocele and the presence of accessory renal artery type and other types as the upper kidney's blood supply artery are independent risk factors for postoperative adverse events.

## Introduction

Duplex kidney is a common genitourinary congenital anomaly in children. It denotes the presence of two distinct collecting systems in one kidney, typically arranged in an upper and lower configuration (1). The upper moiety is frequently associated with developmental abnormalities, with an incidence rate between 0.7% and 0.8% (2). Duplex kidney can be classified into complete and incomplete types. The complete type refers to both ureters opening into the bladder or other locations separately, while the incomplete type refers to the convergence of the two collecting systems before entering the bladder, resulting in a single ureter opening into the bladder (3). In some cases, the diagnosis of duplex kidney is made during late-stage fetal ultrasound examination or through urinary system ultrasound examination conducted for other conditions. Some affected children may present with symptoms such as recurrent urinary tract infections, ureteroceles affecting normal urination, and intermittent involuntary urinary leakage.

There is some controversy surrounding the treatment of duplex kidney (4), but surgical excision is commonly used, particularly for duplex kidney with developmental abnormalities and concurrent clinical symptoms, for which heminephrectomy is the most common surgical procedure. Some children may experience postoperative adverse events, such as recurrent urinary tract infections, residual renal dysfunction, and “perinephric urinoma”, but the majority recover well. There is currently no consensus regarding the factors that contribute to the occurrence of these adverse events. This study's objective is to identify the risk factors for postoperative adverse events in children with duplex kidney undergoing upper pole heminephrectomy.

## Methods

**Figure 1:**
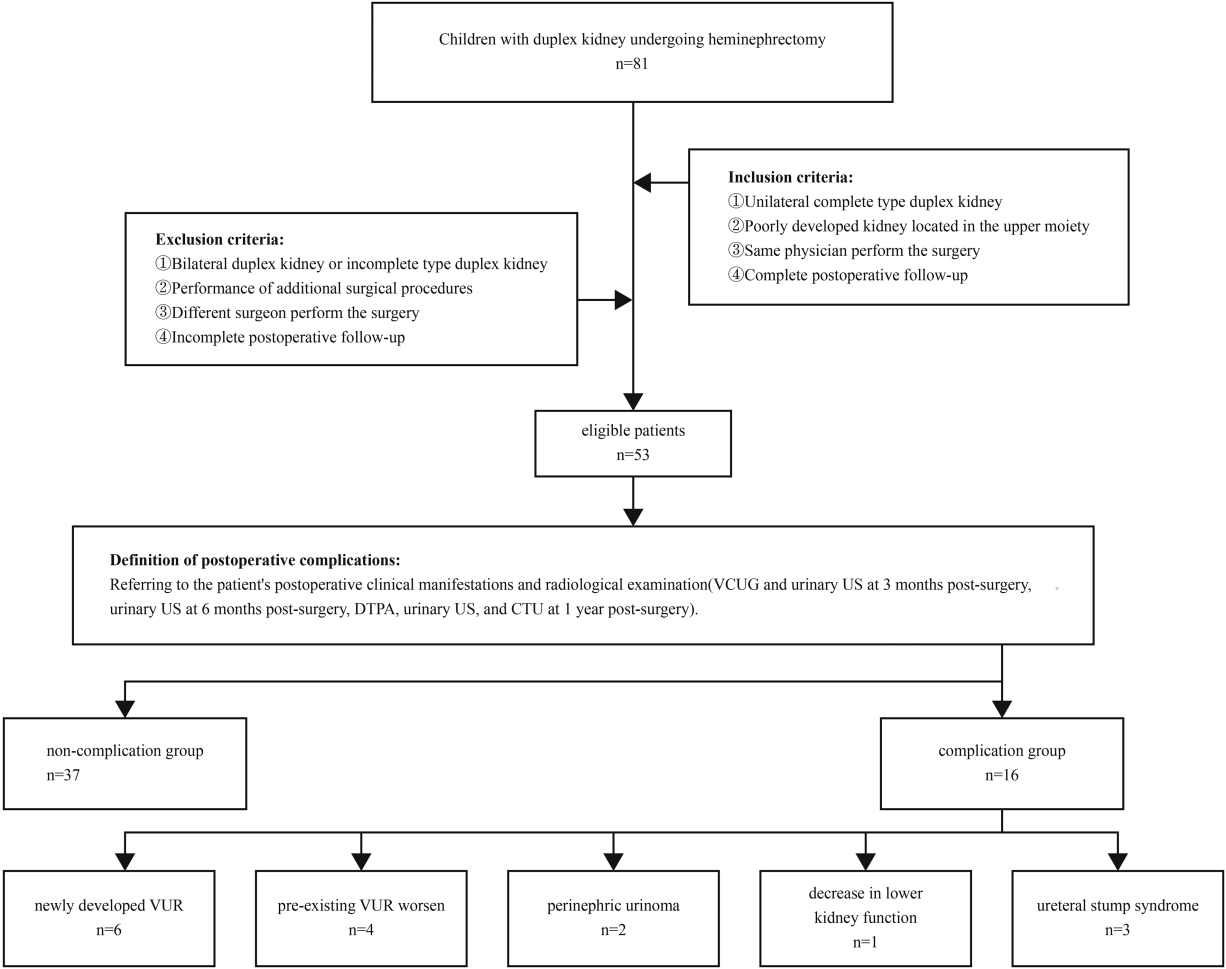
VCUG, voiding cystourethrography; DTPA, diethylenetriaminepentaacetic acid; US, ultrasound; CTU, computed tomography urography; VUR, vesicoureteral reflux.

We collected clinical data from pediatric patients with duplex kidney who underwent upper pole heminephrectomy in the Department of Pediatric Urology at the First Affiliated Hospital of Zhengzhou University between January 2016 and January 2021. We collected clinical data from pediatric patients who met the inclusion criteria and exclusion criteria (Figure 1).

All pediatric patients undergo comprehensive urinary system examinations including ultrasound, intravenous pyelography (IVP), voiding cystourethrography (VCUG), computed tomography urography (CTU), and diethylenetriaminepentaacetic acid (DTPA) renal scintigraphy upon admission. When the split renal function of the upper kidney is less than 10%, it is considered as poor or non-functional development of the upper kidney. Surgical indications for heminephrectomy may include the following symptoms: 1. severe progressive hydronephrosis of the upper kidney 2. ureteral dilation causing bladder outlet obstruction 3. recurrent urinary tract infections. Surgical technique and approach can be divided into two types: laparoscopic heminephrectomy and open heminephrectomy, both procedures are trans peritoneal.

Based on the presence or absence of postoperative adverse events, the patients were divided into two groups: an adverse events group (*n* = 16) and a non-adverse events group (*n* = 37). We collected data on potential factors that may be associated with the occurrence of postoperative adverse events. This study was approved by the Ethics Committee of the First Affiliated Hospital of Zhengzhou University, and all methods were performed in accordance with the relevant guidelines and regulations, all patients or their family members have signed the informed consent before surgery and provided the consent to publish and report individual clinical data. All 53 cases were followed up completely, and regular follow-up was conducted outside the hospital through telephone or online communication to monitor any adverse reactions. The follow-up duration for all patients exceeded 2 years.

**Figure 2:**
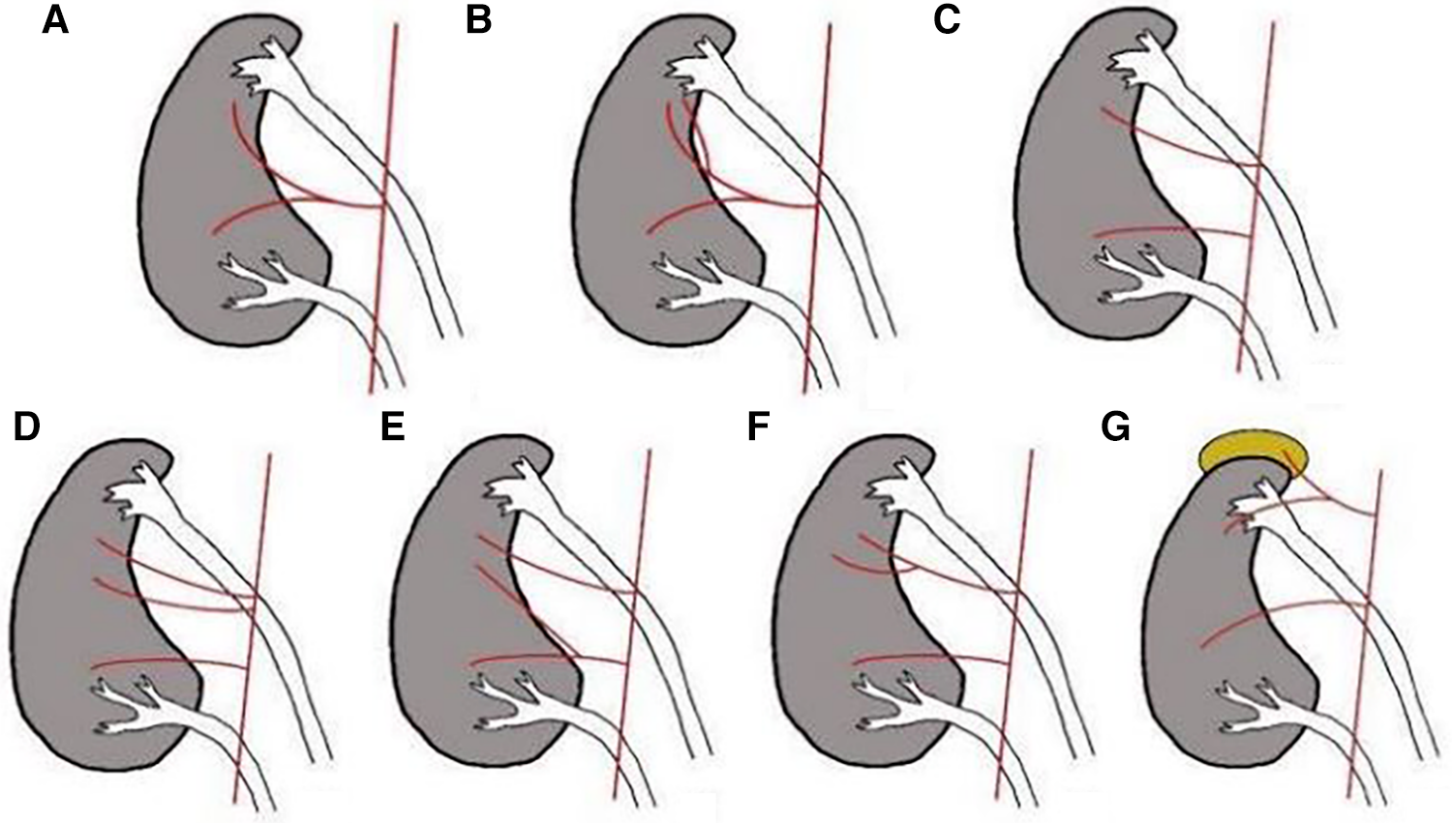
Classification of the blood supply artery to the upper pole kidney. Early branching type: (**A**) The single renal artery branch supplies the upper kidney. (**B**) Multiple renal artery branches supply the upper kidney. Accessory renal artery type: (**C**) The single accessory renal artery supplies the upper kidney. (D) Multiple accessory renal arteries supply the upper kidney. Other types: (**E**) The renal artery branch and the accessory renal artery supply the upper kidney simultaneously. (**F**) Multiple accessory renal artery branches supply the upper kidney. (**G**) Adrenal artery branches supply the upper kidney.

Classification of the blood supply artery to the upper pole kidney (Figure 2): The preoperative CTU images of the patients were collected for three-dimensional reconstruction of the renal vasculature. Based on the origin, course, and opening characteristics of the arterial supply to the upper kidney, classification was performed by referring to the anatomical classification of normal renal arteries and relevant literature.

## Statistical analysis

Statistical analysis was performed using SPSS 26.0 software. For normally distributed continuous variables, an independent samples *t*-test was used for between-group comparisons, with the value of $\bar{x}\pm s$ assigned. If the continuous variables did not follow a normal distribution, an independent samples Mann-Whitney *U*-test was used for between-group comparisons, with the value of M (Q1, Q3) assigned. Qualitative data were analyzed by the *χ*^2^-test and represented as frequencies and percentages [*n* (%)] on both groups. Significant variables identified through screening were subjected to multivariate logistic regression analysis using a binary logistic regression model to determine the odds ratios and 95% confidence intervals for independent risk factors. Statistical significance was set at *P* < 0.05.

## Results

There were 16 occurrences of postoperative adverse events among the 53 pediatric patients, 6 cases newly developed VUR following surgery, with 4 cases occurring on the same side and 2 cases on the opposite side, among these, 3 cases of grade I reflux, 2 cases of grade II reflux, and 1 case of grade III reflux. In addition, 4 cases of pre-existing VUR worsened, with 2 cases progressing from grade I to grade II, 1 case from grade II to grade IV, and 1 case from grade III to grade IV. 2 patients presented with “perinephric urinoma” following surgery. 1 case showed a postoperative decrease in lower kidney function, declining from 38.3% before the surgery to 17.3% after the surgery. 3 cases developed ureteral stump syndrome after surgery, with 2 cases presenting recurrent urinary tract infections accompanied by abnormal urethral discharge and 1 case presenting postoperative protrusion of the renal ureterocele resulting in bladder outlet obstruction (Table 1). Univariate analysis identified age (*P* = 0.014), the presence of upper renal ureterocele (*P* = 0.026), intraoperative injury to the lower kidney (*P* = 0.011), the location of ureteral stump ligation (*P* = 0.024), and the type of blood supply artery to the upper pole kidney (*P* = 0.010) as factors influencing postoperative adverse events (Table 2).

**Table 1:** Postoperative adverse events after upper pole heminephrectomy.

Variable	Case, *n* (%)
Newly developed VUR	
Grade I	3 (5.7)
Grade II	2 (3.8)
Grade III	1 (1.9)
Pre-existing VUR worsened	
Grade I to grade II	2 (3.8)
Grade II to grade IV	1 (1.9)
Grade III to grade IV	1 (1.9)
Perinephric urinoma	2 (3.8)
Lower kidney function decrease	1 (1.9)
Ureteral stump syndrome	3(5.7)

VUR, vesicoureteral reflux.

**Table 2:** Patient characteristics between the PAV and non-PAV groups.

Variable		n-PC group (*n* = 37)	PC group (*n* = 16)	Z/*χ*^2^	*P*-value
Age		2.30 ± 1.21	1.61 ± 0.73	2.552	0.014*
Weight		12.78 ± 2.56	11.51 ± 1.87	1.780	0.081
Preoperative split renal function of the upper kidney		7.67 ± 2.29	7.52 ± 2.52	0.210	0.834
Preoperative split renal function of the lower kidney		38.74 ± 6.09	38.98 ± 6.49	−0.128	0.899
Preoperative parenchymal thickness of the lower kidney		8.14 ± 1.23	8.64 ± 1.46	−0.955	0.349
Gender	Male	15	7	0.047	0.828
	Female	22	9		
Presence of upper renal ureterocele	Yes	6	8	4.936	0.026*
	No	31	8		
Presence of upper kidney VUR	Yes	10	6	0.191	0.662
	No	27	10		
Presence of lower kidney VUR	Yes	14	5	0.211	0.646
	No	23	11		
Surgical approach	Open	10	5	0.098	0.754
	Laparoscopic	27	11		
Presence of intraoperative injury to the lower kidney	Yes	1	4	6.500	0.011*
	No	36	12		
Location of ureteral stump ligation	Above iliac vascular	9	9	5.076	0.024*
	Below iliac vascular	28	7		
Type of blood supply artery to the upper-pole kidney	Early branching type	27	5	8.262	0.010*
	Accessory renal artery type	8	8		
	Other types	2	3		

PAV, postoperative adverse events; n-PAV, non-postoperative adverse events.

* *P* < 0.05.

The univariately identified factors were included in the multivariate analysis, which resulted in the following results: Upper renal ureterocele increased the risk of postoperative adverse events (*P* = 0.042, OR = 7.116, 95% CI 1.073–47.172). In addition, compared to the early branching type as the blood supply artery to the upper kidney, the presence of accessory renal artery type (*P* = 0.016, OR = 10.639, 95% CI 1.551–72.978) and other types (*P* = 0.039, OR = 3.644, 95% CI 0.351–37.836) increases the risk of postoperative adverse events, with these differences being statistically significant (Table 3).

**Table 3:** Multivariate logistic regression analysis of postoperative adverse events (*n* = 53).

Variable	B	SE	*P*-value	OR	OR (95% CI)
Age	−0.649	0.470	0.167	0.522	0.208–1.313
Presence of upper renal ureterocele					
Yes	1.962	0.965	0.042	7.116	1.073–47.172
No	0.000				1.000
Presence of intraoperative injury to the lower kidney					
Yes	2.380	1.530	0.120	10.801	0.539–216.515
No	0.000				1.000
Location of ureteral stump ligation					
Above iliac vascular	1.114	0.831	0.180	3.046	0.597–15.539
Below iliac vascular	0.000				1.000
Type of blood supply artery to the upper pole kidney					
Early branching type	0.000				1.000
Accessory renal artery type	2.365	0.982	0.016	10.639	1.551–72.978
Other types	1.293	1.194	0.039	3.644	0.351–37.836

## Discussion

The reported rates of postoperative adverse events following upper pole heminephrectomy differ marginally. Husmann found an 80% cure rate for upper pole heminephrectomy in one study (4, 5), whereas Le's study revealed that 10% of patients required additional surgical treatment (6). Consistent with other studies, the incidence of postoperative adverse events in this investigation was 30.2% (16/53) and the reoperation rate was 9.4% (5/53).

Using multivariate logistic regression, we determined that the preoperative presence of upper renal ureterocele was a risk factor for postoperative adverse events. Renal ureterocele is a relatively common adverse events in patients with duplex kidney. According to studies, approximately 38% of patients with duplex kidney have associated renal ureterocele, with approximately 80% of cases originating in the upper kidney (7). Previous research indicates that approximately 2.2% of patients with duplex kidney and renal ureterocele who underwent upper pole heminephrectomy require additional surgery to remove the distal stump of the ureter (8). In our investigation, this proportion was 3.7%. There is no uniform standard for the surgical treatment of renal ureterocele in patients with duplex kidney. In addition to upper pole heminephrectomy, it is also possible to perform endoscopic ureterocele decompression, ureterocele excision, and lower ureteral reimplantation simultaneously (9). However, upper pole heminephrectomy remains the standard procedure because most renal ureteroceles tend to atrophy following upper pole heminephrectomy (10). Additionally, endoscopic ureterocele decompression is associated with a higher rate of adverse events, particularly new-onset VUR, occurring in approximately 65% of cases (11). The occurrence of new-onset VUR and the deterioration of preexisting VUR after surgery may be attributable to renal ureterocele. This may be attributed to the fact that the renal ureterocele receives blood supply from both the ureter and the bladder, resulting in incomplete atrophy of the dilation following surgery.

This may be due to the fact that even though the renal ureterocele undergoes atrophy after the removal of the upper kidney and ureter, the defect in the trigone of the bladder persists, potentially leading to the development of new-onset VUR (12). In addition, according to the Weigert-Meyer law, the upper ureter typically opens below the normal lower ureter; therefore, when the upper kidney is affected by renal ureterocele, it may alleviate the lower kidney VUR to some degree. However, after renal ureterocele atrophy, the lower kidney VUR may exacerbate.

Normal renal artery anatomy dictates that each kidney is supplied by a single renal artery, which divides at the renal hilum into anterior and posterior segmental arteries. The variation rate of renal blood vessels in the Chinese population ranges from 30% to 40%, and the variations are categorized as early branching type and accessory renal artery type (13). In cases of duplex kidney, besides the early branching type and accessory renal artery type, there are also other types. The most important aspect of an upper pole heminephrectomy is the precise ligation of the blood supply artery of the upper kidney. The precise ligation of the blood supply artery permits a distinct demarcation between the upper and lower kidneys, which facilitates surgical resection. Simultaneously, comprehensive ligation of the artery also helps reduce the occurrence of postoperative bleeding (14). The postoperative “perinephric urinoma” mainly originates from residual margin secretion following upper pole heminephrectomy, the complete ligation of the blood supply artery to the upper kidney can effectively reduce the secretion function of the residual margin and lower the risk of postoperative “perinephric urinoma”. If the blood supply artery to the lower kidney is mistakenly ligated during the surgery, it can lead to a decrease in postoperative renal function of the lower kidney (15). Before undergoing upper pole heminephrectomy, it is necessary to combine CTA imaging to ascertain the type of upper kidney blood supply artery.

In addition, there is controversy regarding the influencing factor of the location of ureteral stump ligation, which was identified through univariate analysis. The current mainstream opinion for upper pole heminephrectomy is to remove the distal ureter as much as possible, but there are differing views (16). Many scholars believe that a long residual segment of the ureter after surgery is associated with the development of ureteral stump syndrome, as an elongated residual segment may be considered a “diverticulum” of the bladder, increasing the risk of postoperative infection (16, 17). Some researchers, however, argue that preserving a certain length of the ureter may have the peristaltic function, preventing urinary stasis, and that it may not be necessary to entirely remove the upper ureter (18, 19). During the distal dissection of the upper ureter, the upper and lower ureters may exhibit a “common ureteral sheath” phenomenon, in such cases, if only the distal ureter is pursued for removal, it may lead to injury to the lower ureter, increasing the risk of postoperative complications. If there is no “common ureteral sheath” phenomenon between the upper and lower ureters, our experience suggests that a laparoscopic approach is typically utilized, and the distal upper ureter is dissected as far as the bladder before ligation. However, if a “common ureteral sheath” phenomenon is present, every effort is made to dissect the upper ureter below the level of the iliac vessels without injuring the lower ureter. After ligation and transection, the residual mucosa is coagulated using an ultrasonic scalpel or electrocoagulation electrotome to minimize leakage from the remnant.

There are limitations in this study, such as the small number of samples, which may affect statistical efficiency. Additionally, this study is single-center and lacks data support from other centers. We hope to obtain support from other centers in the future to conduct a multi-center study with a larger number of samples.

## Conclusion

Present study has found that, in pediatric patients with duplex kidney undergoing upper pole heminephrectomy, the presence of upper renal ureterocele and the presence of accessory renal artery type and other types as the upper kidney’s blood supply artery are independent risk factors for postoperative adverse events.

## Data Availability

The raw data supporting the conclusions of this article will be made available by the authors, without undue reservation.
